# Enhanced Oxidative Stability of Walnut Kernels by Lipid-Soluble Antioxidant Combinations: From Individual Screening to Ternary Formulation

**DOI:** 10.3390/foods15173075

**Published:** 2026-08-30

**Authors:** Shanshan Jiang, Rongrong Wang, Huiliang Wen, Zihang Fu, Jianhua Xie

**Affiliations:** 1 State Key Laboratory of Food Science and Resources, Nanchang University, No. 235 Nanjing East Road, Nanchang 330047, China; jss19843488269@163.com (S.J.);; 2 College of Food Science and Technology, Nanchang University, Nanchang 330031, China

**Keywords:** walnut kernel, lipid oxidation, oxidative stability, lipid-soluble antioxidant

## Abstract

Walnut kernels are highly susceptible to lipid oxidation because of their high content of unsaturated fatty acids. This study screened five lipid-soluble antioxidants—tert-butylhydroquinone (TBHQ), butylated hydroxytoluene (BHT), propyl gallate (PG), dilauryl thiodipropionate (DLTP), and ascorbyl palmitate (AP), and further evaluated binary and ternary formulations based on BHT. Treated walnut kernels were subjected to accelerated oxidation, and the oxidative stability was assessed using peroxide value, thiobarbituric acid-reactive substances (TBARS), DPPH radical scavenging activity, electron spin resonance, fatty acid composition, thermogravimetric analysis, and sensory evaluation. Among the individual antioxidants evaluated at their respective maximum permitted concentrations, BHT showed the most consistent protective effect, with the lowest oxidation indices, the strongest DPPH radical scavenging activity, and the weakest thermally induced radical signal. The BHT + TBHQ formulation performed significantly better than BHT alone in several oxidation indices (*p* < 0.05). The ternary BHT + TBHQ + DLTP formulation showed the best overall performance, with antioxidant activity comparable to that of the commercial antioxidant blend and significantly lower TBARS values than the BHT + TBHQ group (*p* < 0.05). Linoleic, oleic, and α-linolenic acids remained at 58.78–62.54%, 17.62–21.33%, and 10.64–11.31%, respectively. No significant differences in aroma or flavor were observed among treatments (*p* > 0.05). These findings indicate that BHT + TBHQ + DLTP effectively improves the oxidative stability of walnut kernels during accelerated storage.

## 1. Introduction

Walnut is renowned for its rich nutritional profile, comprising substantial amounts of polyunsaturated fatty acids (PUFAs), protein, vitamin E, and polyphenols, which contribute to various health benefits such as reduced cardiovascular risk, anti-inflammatory effects, and neurodevelopment promotion [1,2,3]. However, the high concentration of unsaturated fatty acids (UFAs) also makes walnuts and their products highly susceptible to lipid oxidation during processing and storage. This leads to rancidity, nutrient degradation, and the formation of potentially harmful compounds, ultimately compromising shelf life, sensory quality, and safety [4,5].

To effectively retard lipid oxidation, the addition of antioxidants is a common and effective strategy in the food industry. Currently used antioxidants primarily include synthetic antioxidants, such as butylated hydroxytoluene (BHT) and tert-butylhydroquinone (TBHQ), as well as natural antioxidants, such as rosemary extract (RE), tea polyphenols (TP), and phytic acid (PA) [6]. These antioxidants interrupt the oxidative chain reaction through various mechanisms, including free radical scavenging, singlet oxygen quenching, or metal ion chelation. However, in complex food matrices, the protective effect of a single antioxidant is often limited due to its specific mechanism of action. Furthermore, given consumers’ growing preference for “clean labels” and strict regulatory limits on the use of synthetic antioxidants, developing efficient and safe antioxidant strategies has become particularly important.

Recently, combining antioxidants with complementary mechanisms has emerged as a promising approach, potentially yielding enhanced antioxidant effects that enhance overall performance and allow for reduced dosages [7]. For instance, complementary interactions between primary antioxidants (e.g., phenolics) and secondary antioxidants (e.g., metal chelators) have been shown to improve oxidative stability in various lipid systems [8]. While prior research has explored antioxidants in generic lipid models or some nuts, a systematic investigation focusing on lipid-soluble antioxidants—particularly their binary and ternary combined formulations—within the specific context of high-UFAs walnut kernels remains insufficient. Existing studies often center on single antioxidants or limited comparisons, lacking a comprehensive framework that spans from individual to combined applications in lipid-soluble systems. Moreover, a robust evaluation requires moving beyond primary oxidation indices (e.g., peroxide value, POV) to integrate multidimensional metrics such as secondary oxidation products (e.g., thiobarbituric acid-reactive substances, TBARS), 1,1-diphenyl-2-picrylhydrazyl (DPPH) radical scavenging activity, electron spin resonance (ESR), fatty acid (FA) profile changes, and thermal stability (TGA) for deeper mechanistic insights.

The present study aimed to develop an effective and customized antioxidant combination strategy for walnut kernels. While previous work [9] examined BHT, TBHQ, DLTP, and PG in walnut oil emulsions, systematic investigation of lipid-soluble antioxidant combinations in whole walnut kernels remains limited. To comprehensively evaluate the oxidative stability, we employed peroxide value (POV) for primary oxidation, thiobarbituric acid-reactive substances (TBARS) for secondary oxidation, DPPH scavenging and electron spin resonance (ESR) for radical activity, along with thermogravimetric analysis and fatty acid profiling. The research will first systematically evaluated the efficacy of a series of individual lipid-soluble (TBHQ, BHT, propyl gallate [PG], dilauryl thiodipropionate [DLTP] and ascorbyl palmitate [AP]) antioxidants. On this basis, the most effective individual antioxidants were selected for further investigation of the enhanced antioxidant performance of their binary and ternary combinations. Through accelerated oxidation tests (60 °C), a comprehensive assessment of the oxidative stability of each treatment group will be conducted using multiple analytical techniques, including POV, TBARS, DPPH radical scavenging rate, free radical activity, FA composition analysis, and thermogravimetric analysis (TGA). Ultimately, by comparing the performance with commercially available antioxidant blends, the best-performing antioxidant combination among those tested will be identified, providing a solid theoretical foundation and an efficient technical solution for inhibiting lipid oxidation in walnuts and extending their shelf life.

## 2. Materials and Methods

### 2.1. Materials

Xinjiang Aksu Thin-Shelled Walnut Kernels were purchased from Fenyang Yuankang Native Produce Co., Ltd. (Fenyang, China). The antioxidants TBHQ (≥99.0% purity), DLTP (≥98.0% purity), PG (≥98.0% purity), BHT (≥99.0% purity), and AP (≥98.0% purity) were sourced from Jiangxi Suihong Bio-Technology Co., Ltd. (Shangrao, China). TP (≥98.0% purity) were obtained from Jiangxi Zenong Tea Chemistry Technology Co., Ltd. (Shangrao, China). A commercially available blended antioxidant containing BHT, TBHQ, and citric acid as the main active components was supplied by Anhui Tianlital Food Technology Development Co., Ltd. (Hefei, China).

The following chemicals were used: Potassium thiocyanate (AR grade), phenolphthalein (indicator grade), 5,5-dimethyl-1-pyrroline N-oxide (DMPO, 97% purity), and iron powder (SP, AR grade) from Shanghai Aladdin Biochemical Technology Co., Ltd. (Shanghai, China); methanol (HPLC grade), n-heptane (HPLC grade), potassium hydroxide (AR grade), and 2-thiobarbituric acid (≥98% purity) from Shanghai Macklin Biochemical Technology Co., Ltd. (Shanghai, China); petroleum ether (30–60 °C, AR grade), n-butanol (AR grade), iron(II) chloride tetrahydrate (AR grade), and ethanol (AR grade) from Xilong Scientific Co., Ltd. (Shantou, China); chloroform (AR grade) from Sinopharm Chemical Reagent Co., Ltd. (Shanghai, China); 30% hydrogen peroxide (30%, AR grade) from Shanghai Habor Chemical Technology Co., Ltd. (Shanghai, China); hydrochloric acid (AR grade) from Shanghai Titan Scientific Co., Ltd. (Shanghai, China); DPPH (1,1-diphenyl-2-picrylhydrazyl) radical (≥97% purity) from TCI (Shanghai, China) Chemical Industry Development Co., Ltd. (Shanghai, China); trihenoin (C21:0 triglyceride, standard grade) and a GLC-463 fatty acid methyl ester (FAME) mix standard (standard reference material) from Nu-Chek Prep, Inc. (Elysian, MN, USA); and toluene (AR grade) from Shanghai Runjie Chemical Reagent Co., Ltd. (Shanghai, China).

### 2.2. Experimental Methods

Three control groups were used in this study: (1) Blank control—walnut kernels immersed in 95% (*v*/*v*) ethanol without added antioxidants; (2) Positive control—walnut kernels treated with the commercially available antioxidant blend (0.06%, *w*/*v*, on a fat-weight basis); (3) Initial state—fresh walnut kernels without any treatment or accelerated oxidation, used as the baseline reference. The blank control was included to account for the effect of the ethanol immersion procedure, while the positive control served as an industry-relevant benchmark.

Lipid-soluble antioxidants, including tert-butylhydroquinone (TBHQ), butylated hydroxytoluene (BHT), propyl gallate (PG), dilauryl thiodipropionate (DLTP), and ascorbyl palmitate (AP), were individually dissolved in 95% (*v*/*v*) ethanol to prepare the antioxidant solutions. For each treatment, 200 g of walnut kernels were used per batch, and all experiments were performed in three independent batches (*n* = 3). Walnut kernels were immersed in the antioxidant solutions at a kernel-to-solution ratio of 1:3 (*w*/*v*), corresponding to 3 mL of solution per gram of walnut kernels.

The concentrations of the individual antioxidant solutions were selected with reference to the maximum permitted usage levels specified in the Chinese National Standard GB 2760-2014, and the final concentrations were confirmed based on preliminary experiments to ensure complete dissolution and effective uptake by the kernels. The concentrations of the soaking solutions were 0.03% (*w*/*v*) for BHT, 0.06% (*w*/*v*) for TBHQ, 0.09% (*w*/*v*) for PG, 0.06% (*w*/*v*) for DLTP, and 0.06% (*w*/*v*) for AP.

For each treatment, the required volume of 95% (*v*/*v*) ethanol was transferred into a beaker and preheated in a thermostatic water bath maintained at 60 °C. After the antioxidant had completely dissolved, walnut kernels were added and immersed for 10 min at 60 °C with gentle stirring to ensure uniform contact between the kernels and the antioxidant solution. Following immersion, the kernels were removed, and excess solution adhering to the surface was gently blotted with filter paper.

Antioxidant combinations were subsequently prepared on the basis of the individual treatment concentrations. For binary formulations, each antioxidant was used at one-half of its concentration employed in the corresponding individual treatment. For ternary formulations, each antioxidant was used at one-third of its concentration employed in the corresponding individual treatment. The commercially available antioxidant blend was prepared at a concentration of 0.06% (*w*/*v*, on a fat-weight basis), which is the manufacturer’s recommended usage level for nut products. Walnut kernels immersed in 95% (*v*/*v*) ethanol without added antioxidants served as the control. Following antioxidant treatment, all samples were subjected to accelerated oxidation at 60 °C. Samples were collected after 5 and 8 days of storage for subsequent analyses.

### 2.3. Accelerated Oxidation and Lipid Extraction

Following the immersion treatment described in Section 2.2, the walnut kernels were subjected to accelerated oxidation. The surface moisture of the treated walnut kernels was blotted dry, after which they were subjected to accelerated oxidation in a forced-air drying (DHG-9623 A, Shanghai Jinghong Laboratory Equipment Co., Ltd., Shanghai, China) oven at 60 °C. Approximately 23 half-kernels (about 50 g) per batch were placed in open glass Petri dishes and oxidized in the oven. Samples for days 5 and 8 were prepared as independent batches to avoid repeated sampling effects. The temperature of 60 °C was chosen based on preliminary experiments to accelerate oxidation within a practical time frame, and the sampling days 5 and 8 were selected to represent the primary and secondary stages of lipid oxidation, respectively. Throughout the incubation period, the positions of the samples were routinely rotated to ensure uniform thermal exposure and to minimize positional bias within the oven.

After the accelerated oxidation period, walnut oil was extracted according to the following procedure. Walnut oil was subsequently extracted following the sample pretreatment method for acid value and peroxide value determination as specified in the Chinese National Standard GB 19300-2014. Briefly, the oxidized kernels were ground into a powder. An aliquot of the powder was combined with petroleum ether (30–60 °C boiling range) in a sealed conical flask and agitated on an orbital shaker (HY-2, Guohua Electric Appliance Co., Ltd., Jiangsu, China) for 1 h. The mixture was then left to extract statically in the dark for 12–14 h at room temperature (25 ± 2 °C) with the container wrapped in aluminum foil to protect from light. The solvent phase was recovered by vacuum filtration, and the petroleum ether was removed using a rotary evaporator (N-1300, Shanghai Ailang Instrument Co., Ltd., Shanghai, China) to obtain crude walnut oil. The crude oil was further clarified by centrifugation to separate any residual moisture or particulate matter. The purified oil was stored under nitrogen at −20 °C until analysis.

### 2.4. Peroxide Value (POV) Determination

POV was determined on the walnut oil obtained from Section 2.3 according to the methods described by Jia et al. [10]. Approximately 0.5 g of walnut oil was accurately weighed into a 10 mL volumetric flask and dissolved in a chloroform-methanol (7:3, *v*/*v*) mixture, which was then used to bring the solution to volume, followed by vortex mixing. A 1.0 mL aliquot of this solution was transferred to a dry 10 mL colorimetric tube. One drop of ferrous chloride solution (3.5 g/L) was added, and the mixture was diluted to the mark with the chloroform-methanol (7:3, *v*/*v*) mixture and vortexed. Subsequently, one drop of potassium thiocyanate solution (300 g/L) was added, and the tube was vortexed again. After allowing the mixture to incubate in the dark at room temperature for exactly 5 min, the absorbance was measured at 500 nm by using a UV-Vis spectrophotometer (TU-1900, Beijing Purkinje General Instrument Co., Ltd., Beijing, China) with the chloroform-methanol (7:3, *v*/*v*) mixture as the blank. The peroxide value was calculated using the following equation:

POV (meq/kg) = [(c − c_0_) × V_1_]/[V_2_ × m × 55.84] × 2

where c is the mass of iron in the sample determined from the standard curve (µg), c_0_ is the mass of iron in the blank determined from the standard curve (µg), V_1_ is the total volume of the diluted sample solution (mL), V_2_ is the aliquot volume taken for measurement (mL), m is the sample mass (g), 55.84 is the atomic weight of Fe, and 2 is the conversion factor.

### 2.5. Thiobarbituric Acid-Reactive Substances (TBARS) Value Determination

TBARS value was determined in accordance with the Chinese National Standard GB/T 35252-2017. Briefly, approximately 150 mg of the oil sample was weighed and dissolved in 1-butanol in a 25 mL volumetric flask, which was then brought to volume with the same solvent. A 5 mL aliquot of this sample solution was mixed with 5 mL of TBARS solution (15% trichloroacetic acid and 0.375% thiobarbituric acid in 0.25 mol/L HCl) in a stoppered test tube. The mixture was vortexed and subsequently heated in a 95 °C water bath for 2 h in a fume hood to facilitate the chromogenic reaction. The absorbance of the solution was immediately measured at 530 nm using a double-beam UV-Vis spectrophotometer (TU-1900, Beijing Puxi General Instrument Co., Ltd., Beijing, China) with a 10 mm pathlength cuvette after cooling to room temperature, with distilled water as the reference. A blank was prepared and treated identically by replacing the sample solution with 5 mL of 1-butanol. The TBARS value was calculated using the following equation:

TBARS (mg MDA/kg) = [(A − B) × 50]/m

where A is the absorbance of the sample solution, B is the absorbance of the blank reagent, m is the sample mass (mg), and 50 is the effective factor derived from diluting the sample to 25 mL with 1-butanol and measuring the absorbance using a 10 mm cuvette.

### 2.6. DPPH Free Radical Scavenging Capacity Determination

DPPH radical scavenging activity was determined according to the method described by Yan et al. [11] with slight modifications with slight modifications. Briefly, the walnut oil was diluted with absolute ethanol to a concentration of 10 mg/mL. Then, 4 mL of this sample solution was mixed with 4 mL of a 0.04 mg/mL DPPH solution in a test tube. The mixture was vortexed thoroughly and allowed to incubate in the dark at room temperature for 30 min. The absorbance was subsequently measured at 517 nm by using a TU-1900 UV-Vis spectrophotometer (Beijing Puxi General Instrument Co., Ltd., Beijing, China), with absolute ethanol serving as the blank. The DPPH radical scavenging rate was calculated using the following equation:

DPPH scavenging rate (%) = [1 − (A1 − A2)/A3] × 100

where A1 is the absorbance of the sample solution (4 mL of oil sample + 4 mL of DPPH solution), A2 is the absorbance of the sample blank (4 mL of oil sample + 4 mL of absolute ethanol), and A3 is the absorbance of the control (4 mL of absolute ethanol + 4 mL of DPPH solution). All absorbance values were measured at 517 nm.

### 2.7. Free Radical Activity Determination

The intensity of carbon-centered radicals in walnut oil was monitored by ESR spectroscopy (ESR200M, Bruker Corporation, Karlsruhe, Germany), following the methods described by Chen et al. [12] and Shen et al. [13]. A spin-trapping assay employing DMPO as the trapping agent—suitable for detecting lipid-derived radicals generated during lipid thermal oxidation (110–180 °C) —was utilized. Specifically, a 3% (*w*/*v*) DMPO solution in toluene was prepared by dissolving 30 mg of DMPO in 1 mL of toluene under light-protected conditions and stored at −20 °C. For analysis, 500 μL of oil was mixed with 50 μL of the DMPO solution in a pressure-resistant tube via vortex (QL-906, Haimen Qilinbel Instrument Manufacturing Co., Ltd., Nantong, China) mixing. The mixture was heated in a sand bath at 165 °C for 20 min. Immediately after heating, the sample was aspirated into a capillary tube, the end of which was sealed with solid glue, and promptly placed into a high-sensitivity resonator for EPR measurement. Spectrometer settings were as follows: center field, 3420 G; sweep width, 100 G; sweep time, 13 s; number of scans, 10; modulation amplitude, 1.000 G. Radical signal intensity was measured after baseline correction.

### 2.8. Fatty Acid Composition Determination

Following the method described by Zhang et al. [14], the FA composition of walnut oil was determined., the FA composition of walnut oil was determined. Precisely 10.00 mg of the oil sample was weighed, then mixed with a C21:0 internal standard solution, n-heptane, and a potassium hydroxide-methanol solution. The mixture was vortexed and centrifuged. The supernatant was collected, filtered, and subjected to gas chromatography analysis.

Analysis was performed using a gas chromatograph (Agilent 8890, Agilent Technologies Co., Ltd., Shanghai, China) equipped with a flame ionization detector (FID) and a CP-Sil 88 capillary column (100 m × 0.25 mm × 0.20 μm, Varian Inc., Palo Alto, CA, USA). Hydrogen was used as the carrier gas. Both the injector and detector temperatures were set at 250 °C, and the sample was introduced in split mode. The sample was introduced in split mode with a split ratio of 50:1. Data acquisition and processing were performed using Agilent OpenLab CDS software (version 2.8). Fatty acid methyl esters (FAMEs) were identified by comparing their retention times with those of authentic standards (GLC-463 FAME mix standard, Nu-Chek Prep, Inc., Elysian, MN, USA). Quantification was carried out using the internal standard method with trihenoin (C21:0 triglyceride) as the internal standard, and the results were expressed as the relative percentage of each fatty acid based on peak area normalization. The column temperature program was as follows: hold at 60 °C for 5 min, increase to 170 °C at 11.5 °C/min and hold for 25 min, then ramp to 200 °C at 5 °C/min and hold for 5 min, and finally increase to 215 °C at 2 °C/min for 20 min. FAs were identified by comparing their retention times with those of authentic standards.

### 2.9. Thermogravimetric Analysis Determination

Thermal stability of the walnut oil lipid was assessed using TGA (TGA 8000, PerkinElmer Instruments, Shelton, CT, USA). A sample of (5 ± 0.5) mg was accurately weighed into a hermetically sealed aluminum crucible with a central pinhole. The analysis was performed under a nitrogen purge gas atmosphere with a temperature program from an initial 30 °C to a final 500 °C at a constant heating rate of 10 °C/min.

### 2.10. Sensory Evaluation of Walnut Kernels

Sensory evaluation was conducted by a panel of 10 trained panelists (5 male and 5 female, aged 22–35 years). All panelists received training on sensory attribute identification and scoring criteria prior to evaluation. Samples were coded with three-digit random numbers and presented in randomized order. Each panelist received approximately 5 g of sample per evaluation. Each sample was evaluated in triplicate (*n* = 3) over two separate sessions. All data are reported as mean ± standard deviation. The sensory evaluation of walnut kernels was conducted using the method described by Yuan et al. [15], with the scoring criteria detailed in Table 1, and the overall score was calculated using Equation:

Overall Score = (Seed Coat Color Score × 15%) + (Kernel Color Score × 15%) + (Peelability Score × 20%) + (Aroma Score × 20%) + (Flavor Score × 30%)

This study protocol did not require approval from an ethics review committee. Prior to conducting the sensory evaluation experiments, we obtained informed consent from all participants and protected their privacy rights.

### 2.11. Data Analysis

All measurements were performed in triplicate using three independent batches of walnut kernels per treatment and time point (*n* = 3). Statistical analysis was conducted using IBM SPSS software 26.0 (IBM Corp., Armonk, NY, USA). At each storage time point, the data were subjected to one-way analysis of variance (ANOVA) to determine whether significant differences existed among treatment groups. When ANOVA indicated a significant difference (*p* < 0.05), Duncan’s multiple range test was subsequently performed as a post-hoc test to identify specific significant differences between individual treatment groups at the same time point. A *p*-value < 0.05 was considered statistically significant for all analyses. All figures were generated using Origin 2018 software (OriginLab Corp., Northampton, MA, USA).

## 3. Results

### 3.1. Effect of Lipid-Soluble Antioxidants on the Oxidative Stability of Walnut Kernels

#### 3.1.1. POV

**Figure 1:**
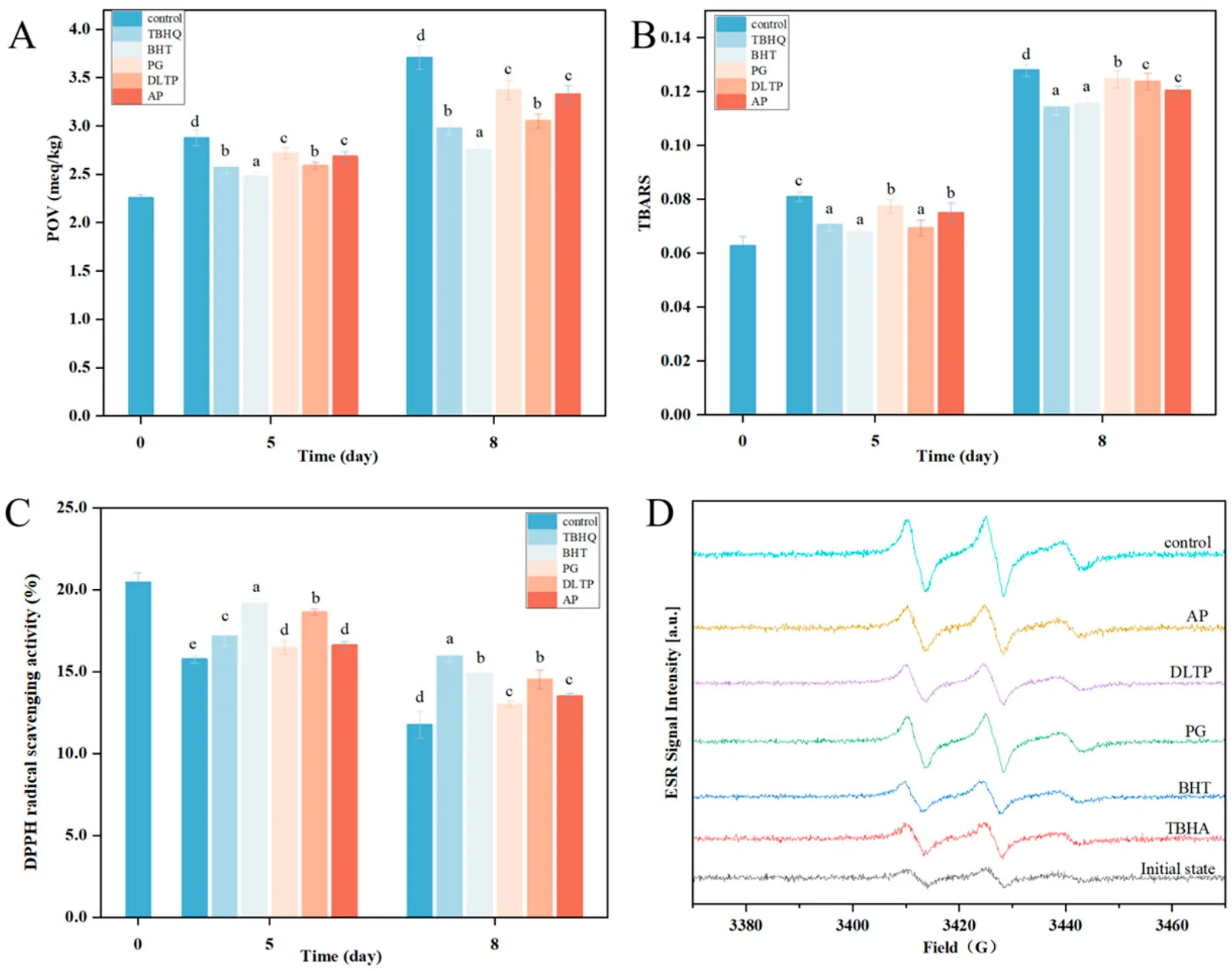
Effects of different lipid-soluble antioxidant treatments on the oxidative stability of walnut kernels. (**A**) Peroxide value (POV), (**B**) Thiobarbituric acid reactive substances (TBARS) value, (**C**) DPPH radical scavenging capacity, (**D**) Electron spin resonance (ESR) spectra. Data are expressed as mean ± standard deviation (*n* = 3). At the same measurement time point, different lowercase letters above the bars indicate significant differences among treatment groups following Duncan’s multiple range test (*p* < 0.05).

POV reflects the formation of primary oxidation products, specifically hydroperoxides, during the initial stage of lipid oxidation. A lower POV indicates less accumulation of hydroperoxides and thus better inhibition of primary oxidation. The effects of different antioxidants and storage time on the POV of walnuts are shown in Figure 1A. As illustrated, the POV increased with prolonged accelerated oxidation time in the oven. However, the rate of increase in POV was slower in groups treated with antioxidants compared to the control group, indicating that the addition of lipid-soluble antioxidants significantly reduced the oxidation of walnuts [16]. After 5 days of oxidation, the effectiveness of the antioxidants was ranked as follows: BHT was the most effective, followed by TBHQ, then DLTP, then AP, and finally PG, with all antioxidant-treated groups performing better than the control. Significant differences (*p* < 0.05) were observed between BHT and TBHQ, DLTP, AP, and PG, as well as between TBHQ/DLTP and AP/PG (*p* < 0.05). After 8 days of oxidation, BHT and TBHQ still demonstrated relatively superior antioxidant efficacy, with BHT showing the most prominent effect. The study by [17] also demonstrated that, compared to other antioxidants, BHT exhibited a more significant inhibitory effect on oxidation, demonstrating superior antioxidant capacity. This POV trend is consistent with the TBARS results discussed below, where BHT also showed the lowest secondary oxidation products. The superior performance of BHT can be attributed to the steric hindrance provided by its two tert-butyl groups, which stabilizes the phenoxyl radical after hydrogen donation and prolongs its radical-scavenging activity.

#### 3.1.2. TBARS Value

TBARS indicate the content of secondary oxidation products, such as aldehydes and ketones, which are primarily responsible for off-flavors and rancidity. TBARS values therefore reflect the extent of secondary lipid oxidation. Figure 1B shows the TBARS values in walnuts following treatment with different lipid-soluble antioxidants. As illustrated, the malondialdehyde (MDA) content increased across all groups with prolonged accelerated oxidation time. This trend can be attributed to the initially low concentration of hydroperoxides, which resulted in limited formation of secondary oxidation products from further oxidative decomposition. After 5 days of oxidation, the efficacy of antioxidants in inhibiting MDA formation was ranked as follows: BHT, DLTP, and TBHQ exhibited comparable efficacy, all of which were more effective than AP; AP, in turn, was more effective than PG, and all antioxidant-treated groups performed better than the control. Among them, BHT showed performance comparable to TBHQ and DLTP but was significantly superior to AP and PG (*p* < 0.05). By day 8 of oxidation, BHT and TBHQ continued to exhibit similar antioxidant activity, and both outperformed PG, DLTP, and AP with statistical significance (*p* < 0.05). These findings demonstrate that the addition of BHT significantly enhances the oxidative stability of walnuts and effectively retards lipid oxidation. The parallel between POV and TBARS results suggests that BHT’s effective scavenging of peroxyl radicals at the primary stage indirectly limited the formation of secondary oxidation products, which are directly associated with sensory deterioration as reflected in the sensory evaluation scores.

#### 3.1.3. DPPH Radical Scavenging Rate

The DPPH radical scavenging assay measures the residual hydrogen-donating capacity of antioxidants, indirectly reflecting their consumption during storage. A higher DPPH free radical scavenging rate indicates stronger antioxidant capacity [18]. The results measured after mixing walnut oil with DPPH reagent and reacting in the dark for 30 min are shown in Figure 1C. As shown in the figure, the scavenging rate was highest at day 0 of accelerated oxidation. With ongoing oxidation, the free radical scavenging ability decreased across all groups, which is likely attributed to the fact that the rate at which antioxidant components scavenged free radicals was lower than the rate of free radical generation. After 5 days of accelerated oxidation, the scavenging rates were ranked as follows: BHT was the highest, followed by DLTP, then TBHQ, then AP, and finally PG. By day 8 of accelerated oxidation, the order was BHT, followed by TBHQ, then DLTP, then AP, and finally PG. BHT demonstrated significantly stronger antioxidant capacity than DLTP, TBHQ, AP, and PG (*p* < 0.05). The decline in DPPH scavenging rates corresponded well with the increases in POV and TBARS, indicating that the consumption of antioxidant capacity directly impacts both primary and secondary oxidation levels. BHT’s sustained scavenging activity explains its consistently lower oxidation indices. It should be noted that walnut oil may contain endogenous antioxidants [13] that contribute to the DPPH scavenging activity. However, since all treatment groups shared the same matrix background, this potential interference was consistent across all samples and did not affect the comparative conclusions among different treatments. Therefore, the reported scavenging rates primarily reflect relative differences rather than absolute antioxidant capacities.

It should also be noted that the DPPH assay measures the residual radical-scavenging capacity of the oil sample, i.e., the ability of remaining antioxidants to directly reduce DPPH radicals, rather than reflecting the actual protection of the oil substrate against oxidation. Furthermore, different antioxidants possess different intrinsic stoichiometric relationships with DPPH due to their distinct molecular structures and reaction mechanisms.

#### 3.1.4. Electron Spin Resonance Pattern

ESR spectroscopy, on the other hand, directly detects carbon-centered free radicals generated during thermal oxidation, providing the most direct evidence of radical formation. Figure 1D displays the ESR spectra of walnut oils from different treatment groups after 8 days of accelerated oxidation. A characteristic signal for carbon-centered radicals, trapped by DMPO upon thermal excitation, was detected in the range of 3400–3450 G. Fresh walnut oil (initial state) showed the weakest signal intensity, serving as the baseline for minimal thermal radical generation. After 8 days of storage, the blank control exhibited a marked increase in signal intensity, indicating that the oil had become highly susceptible to thermally induced radical formation. In contrast, all antioxidant-treated groups exhibited markedly lower signal intensities than the blank control, indicating that the antioxidants conferred residual protection, thereby reducing the oil’s susceptibility to thermal oxidation. Among them, the BHT-treated group displayed the weakest signal, consistent with its superior performance in other antioxidant assays. These results suggest that BHT most effectively preserved the intrinsic stability of the oil matrix against subsequent thermal stress, corroborating the findings from POV and TBARS analyses [19].

#### 3.1.5. Thermogravimetric Analysis

**Figure 2:**
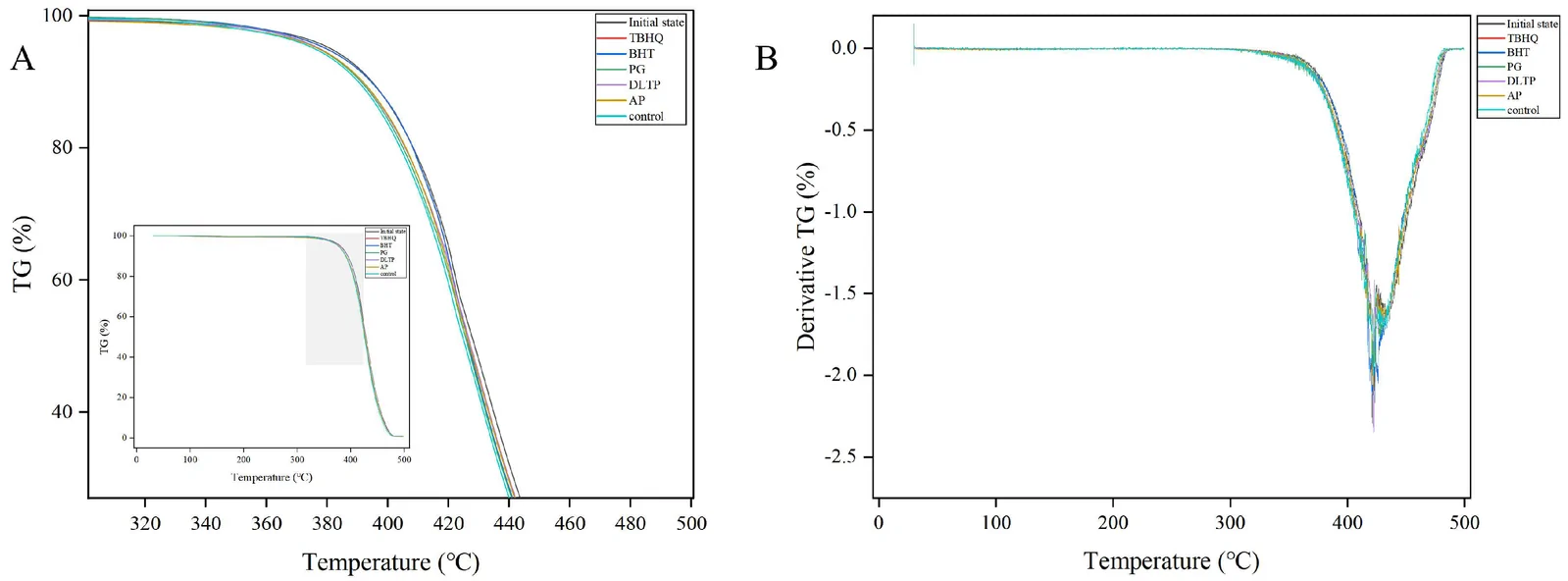
(**A**) Thermogravimetric curves of extracted walnut oil treated with different lipid-soluble antioxidants, (**B**) Derivative thermogravimetric (DTG) curves of extracted walnut oil treated with different lipid-soluble antioxidants.

TGA eflects the thermal stability of the oil, with higher decomposition temperatures indicating better preservation of the oil matrix. The thermal decomposition behavior of walnut oils was evaluated by TGA at a heating rate of 10 °C/min under a nitrogen atmosphere. Representative TG and DTG curves are shown in Figure 2A,B, and the thermal decomposition temperatures (onset temperature, T_5_%, and T_10_%) are summarized in Table 2. The initial walnut oil (before oxidation) exhibited an onset decomposition temperature of 403.898 °C. After 8 days of accelerated oxidation, the decomposition temperatures decreased in all groups, with the control group showing the lowest value (395.006 °C). Among the antioxidant-treated groups, BHT-treated samples showed the highest onset decomposition temperature (401.709 °C), followed by TBHQ (399.521 °C), DLTP (398.016 °C), PG (396.511 °C), and AP (396.511 °C). TGA under a nitrogen atmosphere primarily reflects the thermal stability (pyrolysis resistance) of the oil rather than its oxidative stability. The relatively higher decomposition temperature observed for the BHT-treated samples indicates better preservation of the thermal stability of the oil following accelerated storage.

#### 3.1.6. Sensory Evaluation

**Figure 3:**
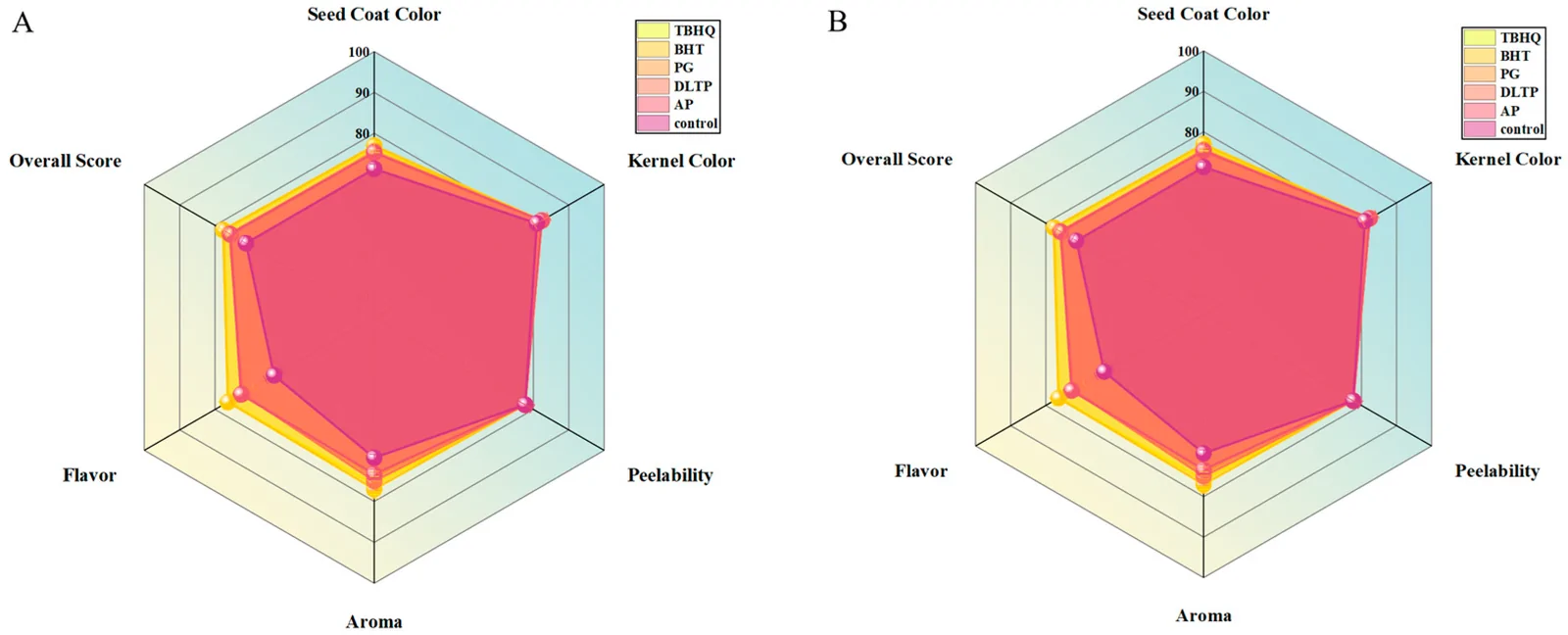
(**A**) Sensory scores of walnut kernels treated with lipid-soluble antioxidants after 5 days, (**B**) Sensory scores of walnut kernels treated with lipid-soluble antioxidants after 8 days. Data are expressed as mean ± standard deviation (*n* = 3).

Sensory evaluation was performed on walnut kernels. The initial scores for non-oxidized kernels were as follows: seed coat color 91, kernel color 92, ease of peeling 89.3, aroma 91.7, flavor 90.7, and overall score 90.9. After 5 days of oven storage, detailed scores are presented in Figure 3A; corresponding results after 8 days are shown in Figure 3B. It was observed that the sensory scores decreased with prolonged storage time in the oven. Although no statistically significant differences were detected in flavor or aroma among the groups, noticeable variations were still present. Numerically, the BHT and TBHQ groups showed the highest mean sensory scores, although these differences were not statistically significant.

Selection of Base Antioxidant for Combinatorial Studies. Based on the comprehensive evaluation above, BHT was selected as the primary antioxidant for subsequent binary and ternary combination studies. While TBHQ and DLTP showed competitive efficacy in specific assays (e.g., TBARS inhibition or DPPH scavenging at certain time points), BHT consistently demonstrated superior or equivalent performance across the most critical indicators of lipid oxidation, notably in maintaining the lowest POV and effectively suppressing secondary oxidation (TBARS) throughout the accelerated storage period. Its balanced efficacy across multiple metrics (POV, TBARS, ESR) justified its selection as the core component for developing effective combinational formulations.

### 3.2. Antioxidant Effects of Binary Lipid-Soluble Antioxidant Combinations

#### 3.2.1. POV

**Figure 4:**
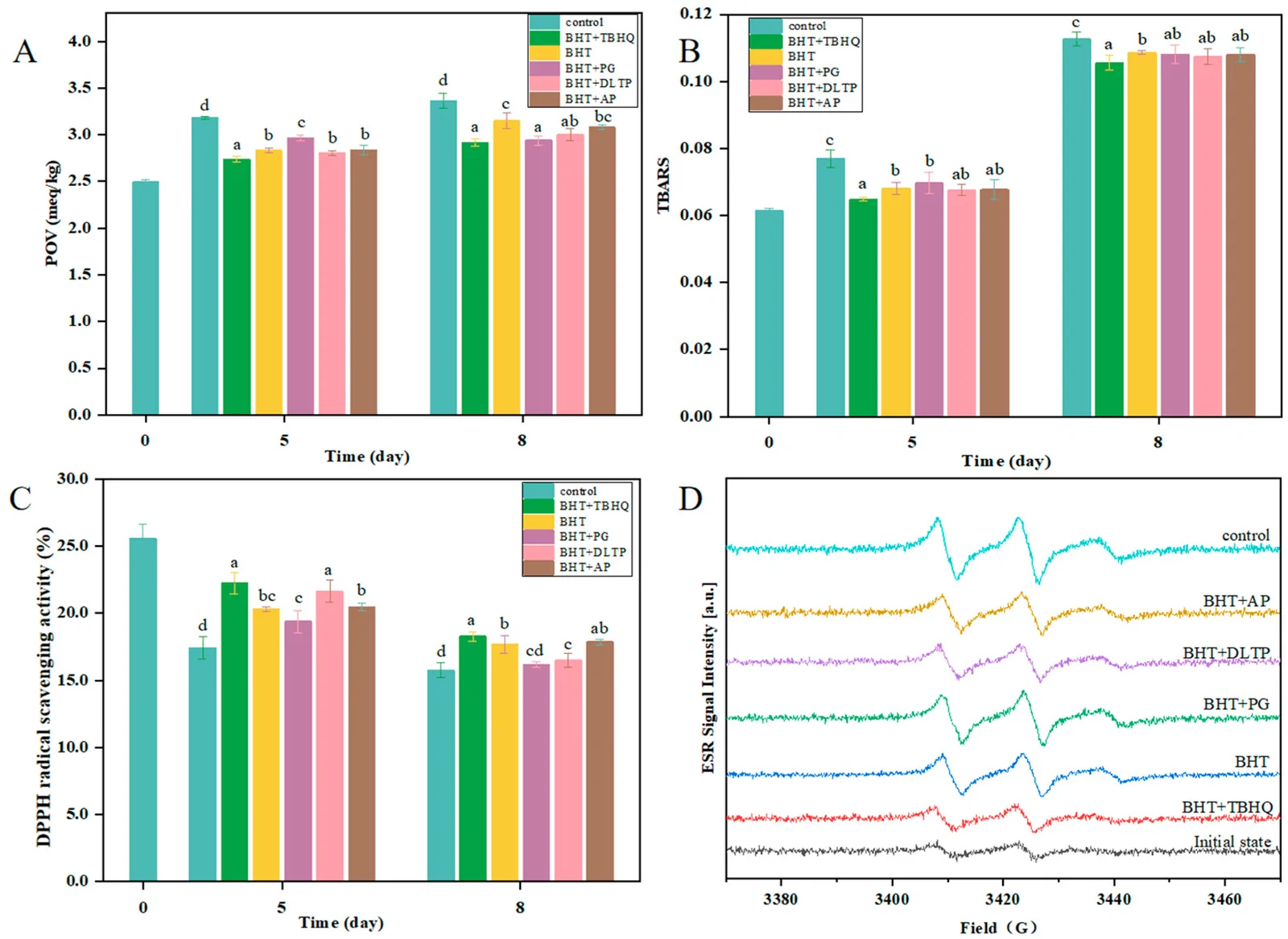
Effects of different binary combinations of lipid-soluble antioxidants on the oxidative stability of walnut kernels. (**A**) Peroxide value (POV), (**B**) Thiobarbituric acid reactive substances (TBARS) value, (**C**) DPPH radical scavenging capacity, (**D**) Electron spin resonance (ESR) spectra. Data are expressed as mean ± standard deviation (*n* = 3). At the same measurement time point, different lowercase letters above the bars indicate significant differences among treatment groups following Duncan’s multiple range test (*p* < 0.05).

The POV progressively increased with extended oven oxidation time in all groups. However, the POVs of the antioxidant-treated groups were consistently lower than that of the control group (Figure 4A), indicating that the antioxidants effectively delayed walnut oxidation. After 5 days of oven oxidation, the antioxidant efficacy was ranked as follows: the combination of BHT and TBHQ was the most effective, followed by BHT combined with DLTP, then BHT alone, then BHT combined with AP, and finally BHT combined with PG, with all treatments being more effective than the control. After 8 days, the order of efficacy was the BHT and TBHQ combination, followed by BHT combined with PG, then BHT combined with DLTP, then BHT combined with AP, then BHT alone, and finally the control. These results indicate enhanced antioxidant performance when BHT was combined with other antioxidants. Notably, the BHT + TBHQ combination performed significantly better than BHT alone. This enhanced efficacy may be attributed to interactions between the antioxidants during their action. Radicals generated from one antioxidant may promote the formation of new phenolic species from another. These newly formed compounds can further contribute to radical scavenging, thus extending the overall protection [20,21].

#### 3.2.2. TBARS Value

As shown in Figure 4B, the initial state of walnut lipids exhibited the lowest TBARS value. Following oven oxidation, a significant increase in TBARS was observed across all groups, which is attributed to the occurrence of secondary oxidation in the walnuts. This trend is consistent with the findings of [22], who also reported a continuous increase in TBARS values in coated walnut samples stored in a 40 °C oven for 12 days. Notably, the TBARS values of the antioxidant-treated groups were significantly lower than those of the control group (*p* < 0.05), confirming the efficacy of the antioxidants in suppressing oxidation. Moreover, the antioxidant capacity of the BHT + TBHQ combination was significantly higher than that of BHT alone, further demonstrating the improved antioxidant efficiency of the BHT + TBHQ combination.

#### 3.2.3. DPPH Radical Scavenging Rate

The DPPH free radical scavenging rates for the different groups are shown in Figure 4C. An overall decreasing trend in scavenging rate was observed with prolonged oven oxidation time. However, the addition of antioxidants significantly mitigated the accumulation of free radicals. After 5 days of oxidation, the BHT + TBHQ and BHT + DLTP combinations exhibited comparable scavenging capacity, which was significantly higher than that of BHT alone (*p* < 0.05), indicating enhanced antioxidant effects between BHT and TBHQ or DLTP. This enhanced effect of the BHT + TBHQ combination was further corroborated after 8 days of oxidation.

#### 3.2.4. Electron Spin Resonance Pattern

Figure 4D displays the free radical signal intensities of walnut lipids across all experimental groups. All groups exhibited signal peaks within the magnetic field range of 3400–3450 G. The initial state of the walnuts showed the lowest radical signal intensity. After oven oxidation, the peak intensity increased, indicating that lipid oxidation had occurred. This observation is consistent with previous studies: [23] reported an increasing trend in radical concentration during the accelerated oxidation of sunflower oil, and [24] also documented significant lipid oxidation accompanied by a rise in radical levels in scallops during accelerated storage. The addition of antioxidants effectively reduced the radical signal intensity, suggesting enhanced residual antiradical activity after storage. Notably, the BHT + TBHQ group exhibited an even lower signal than the BHT group, reflecting its superior residual protective effect against thermally induced radical generation. This observation corroborates the improved antioxidant performance of this binary combination evidenced by the lower POV and TBARS values.

#### 3.2.5. Fatty Acid Composition

**Figure 5:**
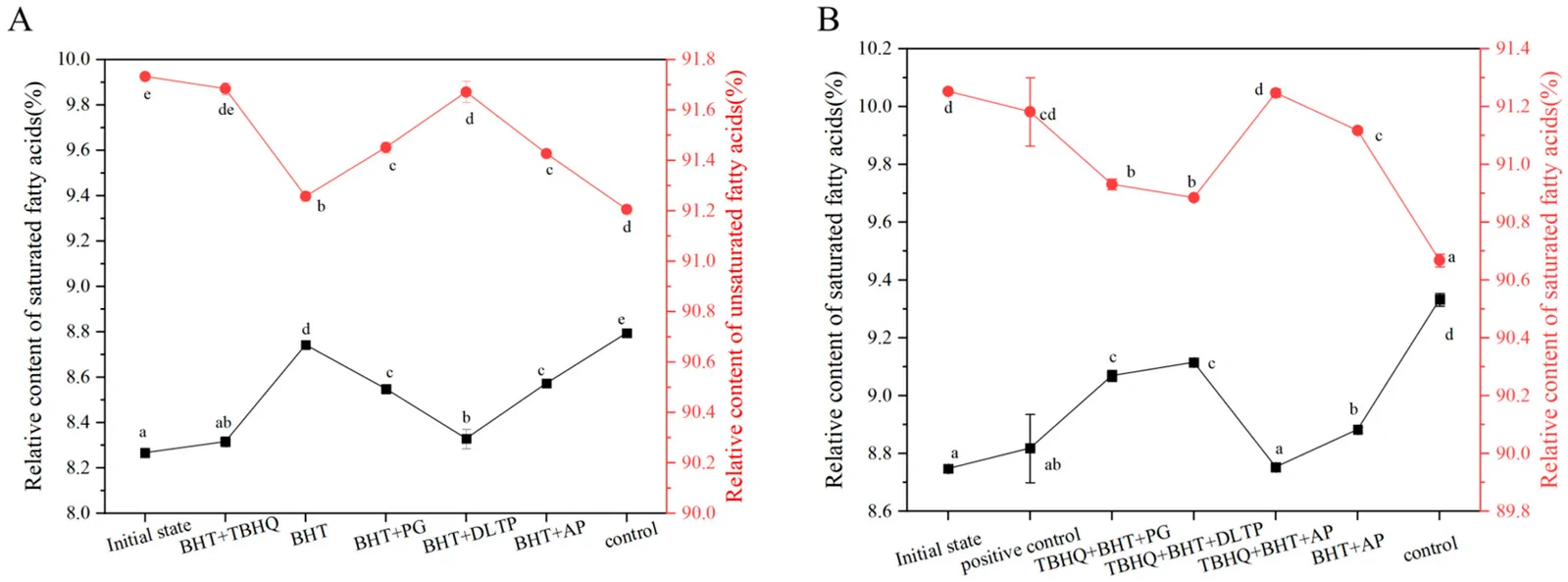
(**A**) Effect of binary combined formulations of lipid-soluble antioxidants on the composition of saturated and unsaturated fatty acids in walnut kernels, (**B**) Effect of ternary combinations of lipid-soluble antioxidants on the composition of saturated and unsaturated fatty acids in walnut kernels. Data are expressed as mean ± standard deviation (*n* = 3). At the same measurement time point, different lowercase letters above the bars indicate significant differences among treatment groups following Duncan’s multiple range test (*p* < 0.05).

The FA compositions of walnut oils after 5 days of accelerated oxidation are presented in Table 3. The proportions of saturated and unsaturated fatty acids for the binary combinations are shown in Figure 5A. Linoleic acid (C18:2), oleic acid (C18:1), and α-linolenic acid (C18:3) remained the predominant fatty acids in all treatments, accounting for 59.13–63.75%, 17.15–21.65%, and 10.42–11.48% of the total fatty acids, respectively. Although statistically significant differences were observed in the relative proportions of several fatty acids among treatments, the characteristic FA profile of walnut oil was generally maintained.

Compared with the control, antioxidant-treated samples showed minor variations in the relative proportions of major fatty acids. It should be noted that because fatty acid compositions are expressed as relative percentages, a higher proportion of linoleic acid is mathematically coupled with a lower proportion of oleic acid due to the sum-to-100% constraint; this does not necessarily indicate greater absolute retention of linoleic acid. However, these differences were relatively small, and no evidence of extensive degradation of unsaturated fatty acids was observed after 5 days of storage.

Unlike peroxide value, TBARS, and ESR, which directly reflect the formation of primary and secondary oxidation products, FA composition mainly reflects structural alterations occurring during relatively advanced stages of lipid degradation. Therefore, FA analysis was not intended to differentiate the antioxidant efficacy of individual treatments but rather to verify whether oxidation had progressed to the stage of substantial FA degradation. The results indicate that walnut oil remained predominantly in the primary and secondary oxidation stages after 5 days of accelerated storage, and the overall FA profiles across all treatment groups remained within the typical range for walnut oil.

Although statistically significant differences were observed in some fatty acids among treatment groups, the overall changes were relatively small and remained within the typical range for walnut oil, consistent with previous studies [1,2]. The relatively small magnitude of these changes within the 5-day accelerated oxidation period suggests that lipid oxidation had not progressed to the stage of extensive unsaturated fatty acid degradation. This observation is consistent with the moderate increases in POV and TBARS values at this time point.

### 3.3. Antioxidant Effects of Ternary Lipid-Soluble Antioxidant Combinations

#### 3.3.1. POV

**Figure 6:**
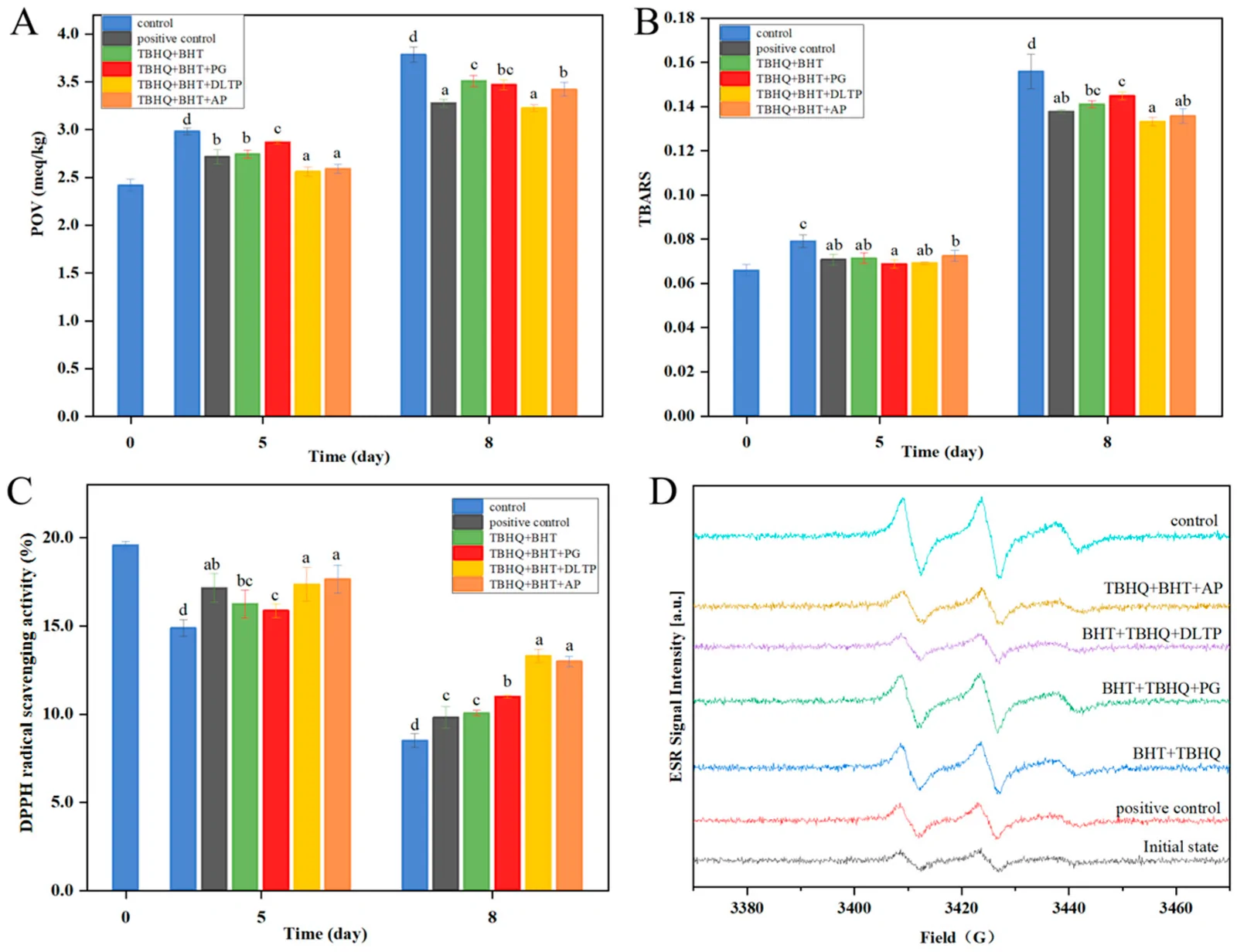
Effects of different ternary combinations of lipid-soluble antioxidants on the oxidative stability of walnut kernels. (**A**) Peroxide value (POV), (**B**) Thiobarbituric acid reactive substances (TBARS) value, (**C**) DPPH radical scavenging capacity, (**D**) Electron spin resonance (ESR) spectra. Data are expressed as mean ± standard deviation (*n* = 3). At the same measurement time point, different lowercase letters above the bars indicate significant differences among treatment groups following Duncan’s multiple range test (*p* < 0.05).

Figure 6A shows the POV of walnut kernels treated with different antioxidants after various periods of oven storage. The POV exhibited an increasing trend with prolonged storage time. Compared with the blank control group, the addition of antioxidants significantly suppressed lipid oxidation and reduced the extent of POV increase. After 5 days of oven storage, the order of antioxidant capacity was ranked as follows: the combination of BHT, TBHQ, and DLTP was the most effective, followed by BHT combined with TBHQ and AP, then the commercial antioxidant, then the combination of BHT and TBHQ, and finally BHT combined with TBHQ and PG, with all treatments outperforming the blank control. This order changed after 8 days to: the BHT + TBHQ + DLTP combination remained the most effective, followed by the commercial blend, then BHT + TBHQ + AP, then BHT + TBHQ + PG, then BHT + TBHQ, and finally the control. These results demonstrate a beneficial combinational effect when the BHT + TBHQ base combination was further supplemented with DLTP, AP, or PG. Notably, the antioxidant capacity of the BHT + TBHQ + DLTP group was comparable to that of the commercial antioxidant group. These findings are consistent with the study by [25], who also observed improved performance in ternary combinations such as BHA + BHT + TBHQ, further supporting the concept of beneficial combinational effects in multi-component antioxidant systems.

#### 3.3.2. TBARS Value

A noticeable decrease in TBARS was observed in antioxidant-treated samples relative to the blank control (Figure 6B), demonstrating successful suppression of lipid oxidation. Following 5 days of oven storage, the BHT + TBHQ + DLTP group showed antioxidant capacity equal to that of the BHT + TBHQ + PG and commercial groups. After 8 days, its performance remained comparable to the BHT + TBHQ + AP and commercial groups, and was significantly stronger than that of the BHT + TBHQ group (*p* < 0.05). This again confirms the beneficial combinational effects among the antioxidants, with DLTP notably aiding the antioxidant performance of the BHT + TBHQ combination in walnuts.

#### 3.3.3. DPPH Radical Scavenging Rate

After 5 days of oven storage, the antioxidant capacity of the BHT + TBHQ + DLTP group was comparable to that of the BHT + TBHQ + AP group and significantly higher than that of the BHT + TBHQ group (*p* < 0.05) (Figure 6C). The same conclusion was reached after 8 days of oven storage. These results confirm the enhanced antioxidant effect of the BHT + TBHQ + DLTP combination. The variation in efficacy among different antioxidants can be explained by the fact that the reaction with DPPH is strongly influenced by the structural conformation of the antioxidant molecules [26]. For phenolic antioxidants, the presence of hydroxyl groups facilitates rapid hydrogen atom transfer to DPPH radicals [27], whereas the antioxidant activity of DLTP is attributed to its thioether structure, which acts through a different mechanism (i.e., hydroperoxide decomposition rather than direct radical scavenging) as discussed in Section 3.3.5.

#### 3.3.4. Electron Spin Resonance Pattern

Figure 6D presents the ESR spectra of free radicals derived from walnuts treated with different antioxidants. The signal peaks for all groups appeared at similar magnetic field strengths, ranging from 3400 to 3450 G, differing only in intensity. The lowest signal intensity was observed in the initial state before oven-induced oxidation, appearing almost as a straight line. After 8 days of oven storage, the signal intensity increased across all groups, indicating accelerated lipid oxidation and continuous generation of free radicals. In contrast, the antioxidant-treated groups showed attenuated signal intensities, reflecting their residual capacity to suppress radical generation upon thermal excitation, likely owing to the retained hydrogen-donating ability of the antioxidants. Among them, the commercial blend, BHT + TBHQ + AP, and BHT + TBHQ + DLTP groups exhibited the lowest signal intensities, indicating the most robust residual protective effects against thermally induced oxidation. These results support the conclusion that the ternary BHT + TBHQ + DLTP formulation, along with the commercial blend, effectively preserves the oxidative resistance of walnut oil even after extended accelerated storage [27,28,29].

#### 3.3.5. Fatty Acid Composition

The FA compositions of walnut oils after 8 days of accelerated oxidation are summarized in Table 4. The corresponding changes for the ternary treatment groups are illustrated in Figure 5B. The initial fatty acid composition of the raw walnut kernels used for the ternary combination experiments is presented as the “Initial state” in Table 4; this initial composition differs slightly from that in Table 3 because the single-antioxidant screening and the ternary combination experiments were conducted using different batches of walnut kernels harvested in the same year from the same cultivar. Linoleic acid (C18:2), oleic acid (C18:1), and α-linolenic acid (C18:3) remained the dominant FAs in all samples, with contents ranging from 58.78–62.54%, 17.62–21.33%, and 10.64–11.31%, respectively. Although statistically significant differences were detected among treatments, the overall FA profile remained largely unchanged after accelerated oxidation. Among all treatment groups, the relative proportions of linoleic acid ranged from 61.17% (control) to 62.54% (BHT + TBHQ + DLTP). These minor variations fall within the typical range for walnut oil and should not be interpreted as evidence of differential preservation of linoleic acid. Because fatty acid compositions are expressed as relative percentages, an increase in the relative proportion of one fatty acid does not necessarily indicate absolute retention; rather, it mathematically reflects the simultaneous decrease in other fatty acids due to the sum-to-100% constraint. Therefore, these relative percentage data should be interpreted as an indication that no extensive degradation of the major unsaturated fatty acids had occurred in any group, rather than as quantitative evidence of preservation [30,31]. These results are consistent with the superior oxidative stability of the best-performing formulation among those tested demonstrated by peroxide value, TBARS, and ESR analyses. FA composition is generally considered an indicator of advanced lipid degradation rather than an index of early oxidation processes. Consequently, it served as supplementary evidence that the best-performing antioxidant combination effectively preserved the overall integrity of the walnut oil matrix during accelerated storage, while oxidation remained primarily at the stage of hydroperoxide formation and secondary oxidation products without causing substantial degradation of the major unsaturated FAs [32].

Although statistically significant differences were observed in some fatty acids among treatment groups, the overall changes remained relatively small and within the typical range for walnut oil [1,2]. The limited changes after 8 days of accelerated oxidation indicate that extensive unsaturated fatty acid degradation had not yet occurred, which correlates with the moderate increases in POV and TBARS values observed at this later time point. The limited changes in the overall fatty acid profiles, particularly in BHT-containing treatments, further support the protective efficacy of the antioxidant formulations by confirming that extensive unsaturated fatty acid degradation had not occurred [13,14].

The superior oxidative stability conferred by the BHT + TBHQ + DLTP ternary formulation can be explained by the complementary mechanisms of these antioxidants. BHT and TBHQ are phenolic primary antioxidants that terminate lipid peroxidation chain reactions by rapidly donating hydrogen atoms to peroxyl radicals (LOO• → LOOH), while forming relatively stable phenoxyl radicals. DLTP is a secondary antioxidant of the thiodipropionate type; its sulfide structure can decompose hydroperoxides into stable alcohols (LOOH → LOH), thereby reducing the formation of secondary oxidation products and potentially regenerating some consumed primary antioxidants. This combination creates a “spatiotemporal complementary” protective network: BHT and TBHQ act rapidly during the initiation and propagation stages of free radical chain reactions, while DLTP acts during the propagation stage to decompose accumulated hydroperoxides. This layered defense mechanism likely accounts for the superior performance of the ternary formulation. Similar combined effects between primary and secondary antioxidants have been reported in other lipid systems [19,20,24].

## 4. Conclusions

In conclusion, this study reveals that while BHT alone exhibits superior efficacy among individual lipid-soluble antioxidants, its combination with TBHQ provided enhanced protection against lipid oxidation compared with BHT alone. The antioxidant performance can be further enhanced through ternary formulations, with the specific combination of BHT, TBHQ, and DLTP identified as the most effective system, delivering protection equivalent to a commercial antioxidant blend. This best-performing ternary system effectively suppresses the formation of primary and secondary oxidation products, as evidenced by the limited changes in fatty acid composition (which remained within the typical range for walnut oil) and the overall stability of the oil matrix during accelerated storage. The findings provide an efficient technical strategy for inhibiting lipid oxidation in walnut kernels, which shows promise for application in retarding lipid oxidation in walnut-based products. Future studies incorporating kinetic modeling, such as Arrhenius or Q10 extrapolation, would be valuable to establish quantitative relationships between accelerated oxidation conditions and real-time shelf-life under ambient storage.

## Figures and Tables

**Table 1:** Scoring criteria for sensory quality indicators of walnut kernels.

Trait	Scoring Criteria				
	90~100 Points	80~89 Points	70~79 Points	60~69 Points	50~59 Points
Seed Coat Color	Light bright yellow	Dark yellow	Dark brown	Brown	Blackish-brown
Kernel Color	White	Yellowish-white	Yellow	Tan	Blackish-brown
Ease of Shelling	Easy	Relatively Easy	Difficult	Difficult	Very difficult
Aroma	Rich and aromatic	Aromatic	Lightly aromatic	Faint and subtle	No aroma
Flavor	Strong walnut flavor, fragrant and crispy	Moderate walnut flavor, crispy	Mild walnut flavor, somewhat crispy	Weak walnut flavor, slightly stale	Slight rancid or sour off-flavor

**Table 2:** Thermal decomposition temperatures of walnut oils treated with different lipid-soluble antioxidants under nitrogen atmosphere.

Group	Oxidation Decomposition Temperature/(°C)	T5%/(°C)	T10%/(°C)
Initial state	403.898	381.24	394.28
TBHQ	399.521	379.53	392.51
BHT	401.709	379.86	394.59
PG	396.511	377.7	390.83
DLTP	398.016	379.53	391.64
AP	396.511	377.15	391.43
Control	395.006	375.79	390.37

Note: “Initial state” refers to fresh walnut kernels without any antioxidant treatment or accelerated oxidation. “Control” refers to walnut kernels subjected to accelerated oxidation without antioxidant treatment. T5% and T10% denote the temperatures at which 5% and 10% of the sample mass are lost, respectively, during thermogravimetric analysis (TGA). These parameters are widely used as indicators of the onset and early progression of thermal decomposition in oil samples.

**Table 3:** Effect of binary combinations of lipid-soluble antioxidants on the fatty acid composition of walnut kernels.

Group	C16:0 (%)	C18:0 (%)	C18:1 (%)	C18:2 (%)	C20:0 (%)	C18:3 (%)
Initial state	5.581 ± 0.00 ^a^	2.609 ± 0.01 ^b^	18.308 ± 0.03 ^c^	62.515 ± 0.02 ^e^	0.077 ± 0.00 ^a^	10.910 ± 0.02 ^d^
BHT + TBHQ	5.589 ± 0.01 ^a^	2.645 ± 0.01 ^c^	19.547 ± 0.04 ^e^	60.655 ± 0.04 ^b^	0.083 ± 0.00 ^c^	11.482 ± 0.03 ^f^
BHT	5.575 ± 0.01 ^a^	3.084 ± 0.01 ^f^	17.477 ± 0.03 ^b^	62.723 ± 0.07 ^f^	0.084 ± 0.00 ^c^	11.058 ± 0.03 ^e^
BHT + PG	5.671 ± 0.02 ^b^	2.794 ± 0.00 ^d^	19.736 ± 0.02 ^f^	60.963 ± 0.02 ^c^	0.084 ± 0.00 ^c^	10.753 ± 0.02 ^c^
BHT + DLTP	5.771 ± 0.02 ^c^	2.480 ± 0.02 ^a^	18.820 ± 0.03 ^d^	61.992 ± 0.09 ^d^	0.078 ± 0.00 ^a^	10.860 ± 0.03 ^d^
BHT + AP	5.825 ± 0.02 ^d^	2.668 ± 0.00 ^c^	17.147 ± 0.04 ^a^	63.750 ± 0.05 ^g^	0.080 ± 0.00 ^b^	10.531 ± 0.02 ^b^
Control	5.753 ± 0.00 ^c^	2.950 ± 0.02 ^e^	21.647 ± 0.02 ^g^	59.134 ± 0.01 ^a^	0.091 ± 0.00 ^d^	10.424 ± 0.02 ^a^

Data are expressed as mean ± standard deviation (*n* = 3). For the same measurement time point, different lowercase letters in the table indicate that there are significant differences (*p <* 0.05) among treatment groups, as determined by Duncan’s multiple range test.

**Table 4:** Fatty acid composition of walnut oils with ternary antioxidant combinations.

Group	C16:0 (%)	C18:0 (%)	C18:1 (%)	C18:2 (%)	C20:0 (%)	C18:3 (%)
Initial state	5.928 ± 0.01 ^a^	2.739 ± 0.00 ^b^	19.614 ± 0.00 ^d^	60.599 ± 0.01 ^d^	0.081 ± 0.00 ^a^	11.039 ± 0.01 ^d^
Positive control	5.901 ± 0.01 ^a^	2.829 ± 0.04 ^d^	21.332 ± 0.05 ^g^	58.779 ± 0.19 ^a^	0.089 ± 0.01 ^b^	11.072 ± 0.02 ^e^
TBHQ + BHT + PG	6.190 ± 0.02 ^d^	2.797 ± 0.01 ^cd^	19.349 ± 0.01 ^c^	60.638 ± 0.02 ^d^	0.083 ± 0.00 ^ab^	10.943 ± 0.00 ^c^
TBHQ + BHT + DLTP	6.250 ± 0.01 ^d^	2.785 ± 0.01 ^c^	17.618 ± 0.02 ^a^	62.545 ± 0.02 ^f^	0.080 ± 0.00 ^a^	10.722 ± 0.01 ^b^
TBHQ + BHT + AP	5.989 ± 0.01 ^b^	2.682 ± 0.00 ^a^	20.478 ± 0.03 ^f^	60.128 ± 0.06 ^c^	0.082 ± 0.00 ^ab^	10.641 ± 0.01 ^a^
BHT + TBHQ	6.093 ± 0.01 ^c^	2.708 ± 0.00 ^ab^	20.06 ± 0.01 ^e^	59.744 ± 0.02 ^b^	0.082 ± 0.00 ^ab^	11.313 ± 0.01 ^f^
Control	6.213 ± 0.02 ^d^	3.034 ± 0.01 ^e^	18.422 ± 0.02 ^b^	61.171 ± 0.05 ^e^	0.086 ± 0.00 ^ab^	11.075 ± 0.02 ^e^

Data are expressed as mean ± standard deviation (*n* = 3). For the same measurement time point, different lowercase letters in the table indicate that there are significant differences (*p <* 0.05) among treatment groups, as determined by Duncan’s multiple range test.

## Data Availability

The original contributions presented in this study are included in the article. Further inquiries can be directed to the corresponding authors.
